# Supplementary material for: Extracting neuronal activity signals from microscopy recordings of contractile tissue using B-spline Explicit Active Surfaces (BEAS) cell tracking
Source: Sci Rep. 2021 May 25;11:10937. doi: 10.1038/s41598-021-90448-4 (PMC8149687; doi:10.1038/s41598-021-90448-4)
Supplement: Supplementary file 1 — Supplementary Information 1. [file 41598_2021_90448_MOESM1_ESM.docx]

**Extracting neuronal activity signals from microscopy recordings of contractile tissue using B-spline Explicit Active Surfaces (BEAS) cell tracking**

**Youcef Kazwiny^1^, João Pedrosa^2,3^, Zhiqing Zhang^1^,**

**Werend Boesmans^4,5^, Jan D’hooge^2^, Pieter Vanden Berghe^1^**

^1^Laboratory for Enteric NeuroScience (LENS), Translational Research Center for Gastrointestinal Disorders (TARGID), University of Leuven (KU Leuven), Leuven, Belgium

^2^Laboratory of Cardiovascular Imaging and Dynamics, Department of Cardiovascular Sciences, University of Leuven (KU Leuven), Belgium

^3^Institute for Systems and Computer Engineering, Technology and Science, INESC TEC, Porto, Portugal

^4^Department of Pathology, GROW-School for Oncology and Developmental Biology, Maastricht University Medical Center, Maastricht, The Netherlands.

^5^Biomedical Research Institute (BIOMED), Hasselt University, Hasselt, Belgium.

*Correspondence: Pieter Vanden Berghe, pieter.vandenberghe@kuleuven.be

**Supplementary information**

1. **Implementation details and polar coordinates**

Unless stated differently, all analyses were performed with the following parameter values: ρ = 32, B-spline scale = 2, matrix size (number of nodes) = 32, competition term weight $w_{c}=1.2$, curvature penalty weight $w_{\kappa}=1$ number of iterations = 200, minimum number of iterations = 50.

During implementation, polar coordinates were chosen as coordinate systems for individual contours because of their efficiency and suitability for closed shape representation. The center of the individual polar representation of a contour is chosen as the geometrical center of gravity (CG) of the contour. It is an intrinsic limitation of polar coordinate systems to represent shapes that do not enclave the origin of the coordinates. During cell tracking, cells can move substantially and therefore we implemented a coordinate re-alignment between timepoints (frames). As a result, the new CG is calculated between frames and assigned as the origin of new coordinates, and interpolation is used to obtain the contour parameters in the new coordinate system (Suppl. Fig. 1).

**Suppl. Figure 1: Contour representation in polar coordinates.** Contour (bold red line) after segmentation in frame f (left) and coordinate translation to the center of gravity (CG, crossed circle) of the object in preparation for the next frame (f+1) (right).


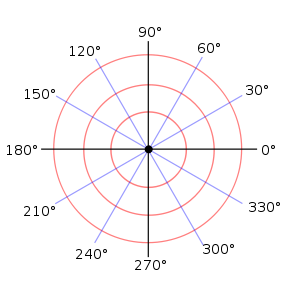

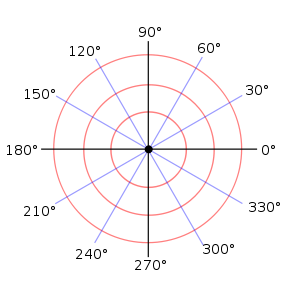


This approach lets cell contours move freely between timepoints, while contour evolution in a specific frame is still limited by the coordinate system. Displacement of cells can be correctly delineated with contours as long as it is less than the cell diameter.

1. **Dataset generation and acquisition**

a/ The artificial dataset


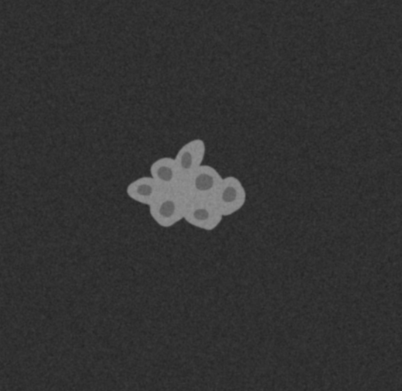
An artificial recording of 7 cells was created to replicate cell shape, size, texture and overlap of cells in ENS recordings of GCaMP expressing cells including the fact that nuclei appear dark. All cells were given the same texture and baseline values, one cell is programmed to stay at its baseline fluorescence while the others were assigned one peak at different times during the recording. A translation movement was applied with similar characteristics as those present in experimental ENS recordings. ImageJ functions were used to add realistic noise and blur levels similar to widefield recordings. We first used “add specific noise” at 10 standard deviation to simulate Poisson noise, after which we added a gaussian blur at 1.2 radius. (Suppl. movie 1) (Suppl. fig 2)

**Suppl. Figure 2: First frame of an artificial dataset**, with 7 cells representing moving, overlapping neurons (full sequence available as Suppl. movie 1).

*b/ The experimental recordings*

Experimental recordings were obtained from several different enteric preparations from Wnt1|GCaMP3 and Wnt1|GCaMP6f mice using multiple microscopy approaches, including widefield (upright and inverted) recordings and spinning disk confocal recordings at 1.5 Hz. For the comparison between manually-drawn ROIs and our methods (Fig. 7 A, B), we used datasets of widefield recordings as described in table 1:

| Dataset | Recording | Genotype | Activity type and desc. |
| --- | --- | --- | --- |
| 1-layer vs manual ROI comparison | 2 Hz | Wnt1xGCaMP3 | chemical (High K^+^ KREBS) stimulation – sudden change in intensities |
|  | 2 Hz | Wnt1xGCaMP3 | chemical (High K^+^ KREBS) stimulation- sudden movement |
|  | 5 Hz | Wnt1xGCaMP6f | Spontaneous activity with tissue movement |
| 2-layer vs manual ROI comparison | 10 Hz | Wnt1xGCaMP6f | chemical (High K^+^ KREBS) stimulation – sudden change in intensities and movement |
|  | 10 Hz | Wnt1xGCaMP6f | chemical (High K^+^ KREBS) stimulation – sudden change in intensities |
|  | 2 Hz | Wnt1xGCaMP3 | chemical (High K^+^ KREBS) stimulation – sudden change in intensities |

Table 1: Ca^2+^ imaging recordings datasets used in the comparison study

*c/ Ground truth peaks spiked in experimental data*

In order to accurately assess the quality of the signal extraction using our newly developed method, we spiked actual experimental recordings with a real and fully described (ground truth) transient Ca^2+^ rise. To guarantee that this rise was as close to reality as possible, a model Ca^2+^ peak was extracted from experimental data using classic registration + ROI selection by an experienced lab member.

We used experimental recordings where the Affine registration method in ImageJ plugin “register virtual slices” was able to completely correct for the motion. Then we manually delineated multiple cells in the registered stack to create a binary mask, which we subsequently blurred (using a gaussian at r=1) to have realistic and non-sharp edges. Custom code in MATLAB was then used to add the model peak intensity values on top of the pixel intensity values in the mask pixels after overlaying it on the recording, producing a recording with natural features and one artificial and fully known activity event in multiple cells. Custom Java code utilizing the ImageJ plugin “TransformJ” was used to perform a “de-registration” and bring back the exact movement of the original stack.

Cell tracking using the newly developed method was performed and the signal was extracted from pixels within the contours. Then we subtracted the baseline (pixel intensity in the mask pixels of the recordings) and compared the signals to the original model peaks using root mean-square error (RMSE) without normalization as the baseline was fully known and the artificial spike amplitude is identical in all cells. For the comparison of our method to traditional ROI selection, where the ground truth is not known and the amplitudes of the extracted signals can vary between methods, we normalized signals before calculating root mean-square error (RMSE).

1. **Comparison between one- and two-layer implementations**

The initialization strategy used between frames is based on the segmentation result from the previous frames (Fig 2) and is the most common and efficient method of initialization. However, the edge-based element in the energy function, can also have, due its force to find edges in the local region, have destabilizing effects. Indeed, if movement between frames exceeds the neighborhood radius ρ, the local search for an edge will fail and the force to stay local will only have a net destabilizing effect on the segmentation. Therefore, the maximum cell movement between frames expected to be tracked successfully is less than ρ, which is therefore an essential parameter that depends on the segmentation strategy chosen. In the double contour strategy, ρ is limited to the nucleus size, whereas it can be increased up to an approximate radius of the cell during one-layer segmentation, which proves to be advantageous in cases with larger displacement.

The installer can be found online:

<https://1drv.ms/u/s!Anfe83lIFXlukrtmWBRy8UQUUjtANg?e=uy7Xt4>


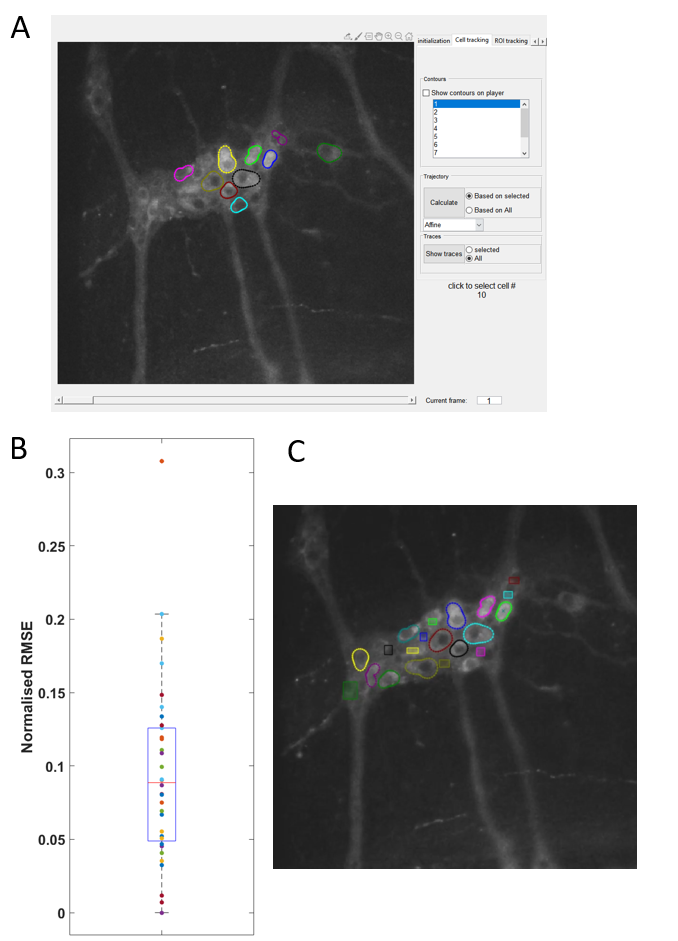


**Suppl. Figure 3: The interface of the cell tracking package.** Several tabs allow initialization, parameter setting, visualization, ROI tracking and trace visualization. A possibility to perform landmark-based ROI tracking is also available.
